# Supplementary material for: Quantum coherent manipulation and readout of superconducting vortex states
Source: Nature. 2026 May 6;653(8113):63–7. doi: 10.1038/s41586-026-10441-7 (PMC13149009; doi:10.1038/s41586-026-10441-7)
Supplement: Supplementary file 1 — This Supplementary Information file has the following sections: I. Set-up and magnetic-field calibration; II. Repeated initialization of VQs; III. Rabi oscillation and active-state preparation; IV. Dispersive measurements; V. Asymmetric quantum Rabi model; VI. Extended time-domain characterization and quantum jumps of VQs; VII. Magnetic-field dispersion from a vortex tunnelling model; VIII. VQ–resonator interaction; IX. Vortex–vortex Interaction; and References. [file 41586_2026_10441_MOESM1_ESM.pdf]

nature portfolio

https://doi.org/10.1038/s41586-026-10441-7

Supplementary information

Quantum coherent manipulation and readout of superconducting vortex states

In the format provided by the
authors and unedited

Supplementary Information for
Quantum Coherent Manipulation and Readout of Superconducting Vortex States

Ameya Nambisan,[1] Simon Günzler,[1, 2] Dennis Rieger,[1, 2] Nicolas Gosling,[1] Simon Geisert,[1] Victor Carpentier,[1] Nicolas Zapata,[1] Mitchell Field,[1] Milorad V. Milošević,[3] Carlos A. Diaz Lopez,[4] Ciprian Padurariu,[4] Björn Kubala,[5, 4] Joachim Ankerhold,[4] Wolfgang Wernsdorfer,[1, 2] Martin Spiecker,[1, 2] and Ioan M. Pop[1, 2, 6, *]

[1] *IQMT, Karlsruhe Institute of Technology, 76131 Karlsruhe, Germany*
[2] *PHI, Karlsruhe Institute of Technology, 76131 Karlsruhe, Germany*
[3] *Department of Physics, University of Antwerp, B-2020 Antwerp, Belgium*
[4] *Institute for Complex Quantum Systems and IQST, University of Ulm, 89069 Ulm, Germany*
[5] *German Aerospace Center (DLR), Institute of Quantum Technologies, 89081 Ulm, Germany*
[6] *Physics Institute 1, Stuttgart University, 70569 Stuttgart, Germany*

CONTENTS

I. Setup & Magnetic Field calibration 2

II. Repeated initialization of VQs 3

III. Rabi Oscillation and Active State Preparation 3

IV. Dispersive measurements 3

V. Asymmetric Quantum Rabi Model 8

VI. Extended Time Domain Characterization and Quantum Jumps of VQs 9

VII. Magnetic field Dispersion from a Vortex Tunneling Model 10

VIII. VQ-resonator Interaction 11

IX. Vortex-Vortex Interaction 11

References 13

* ioan.pop@kit.edu

I. SETUP & MAGNETIC FIELD CALIBRATION

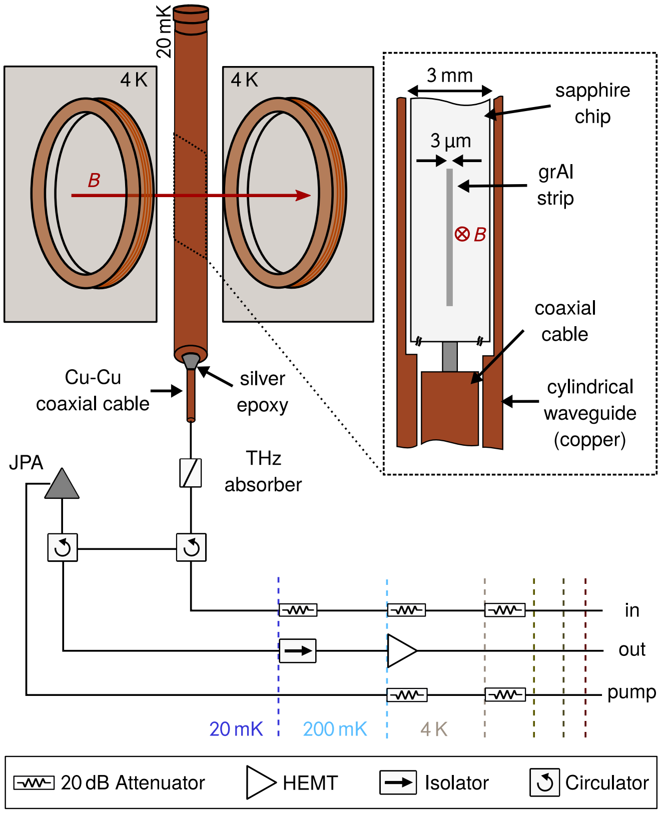

Figure S1. **Measurement setup.** Schematic of the cylindrical copper waveguide sample holder anchored to the millikelvin stage. A Helmholtz coil pair, thermalized to the 4 K stage of the cryostat, generates a perpendicular magnetic field B. The sample holder, directly connected to a THz absorber [1], is measured in single-port microwave reflection S_{11}, with a Josephson parametric amplifier (JPA) [2] on the output line. The input line (marked 'in') is attenuated by 75 dB (including the attenuation of the lines) distributed at the different temperature stages, while the output ('out') is routed through a double-junction isolator and a high electron mobility transistor (HEMT) amplifier. **Inset:** A 3×10 mm sapphire chip is secured with a copper dowel and hosts a grAl microstripline resonator (gray rectangle) positioned 0.5 mm from the bottom edge of the chip, similar to Refs. [3, 4].

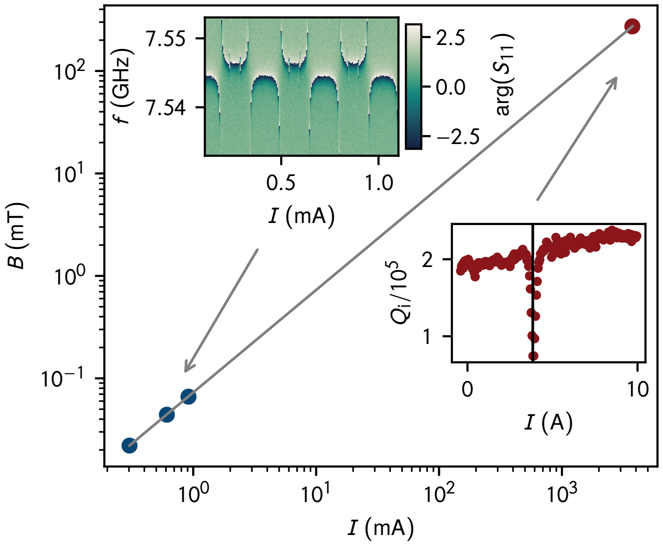

Figure S2. **Magnetic field calibration.** Extracted field B at the sample position versus bias current applied to the Helmholtz coil. For fields up to 100 μT, the flux periodicity (flux sensitivity of 22 μT/Φ_0) of a Gralmonium flux qubit [5] is used for the calibration. The blue markers denote fields corresponding to integer multiples of Φ_0 in the qubit loop. At higher fields, calibration relies on electron spin resonance (ESR) of $g = 2$ spin-1/2 paramagnetic impurities (red marker). A linear fit to both data sets yields a conversion factor of 72.8 mT/A. **Top inset:** Phase response arg(S_{11}) of the readout resonator plotted as a function of the drive frequency f at different bias currents I for the gralmonium qubit described in Ref. [5]. The avoided level crossings appear at field values where the qubit mode periodically crosses the resonator frequency. **Bottom inset:** Internal quality factor Q_i of a grAl resonator versus current I in the coil. A dip, marked by the black line, appears at the current corresponding to ESR between the $g = 2$ spins and the resonator.

Figure S1 shows the cylindrical sample holder with a 3 mm diameter and a 60 GHz cut-off frequency. The resonator is coupled to the microwave readout and control electronics via the evanescent field of the coaxial pin, as illustrated in the inset of Fig. S1. We attribute the elevated VQ temperature of $T_\mathrm{eff} = 74$ mK (cf. Fig. 2a) to thermal photons leaking into the sample holder through the copper dowel, likely due to the absence of additional infrared shielding. This interpretation is supported by similarly elevated temperatures observed in a grAl fluxonium qubit measured in the same setup [4]. The measurements presented in the main text were performed without magnetic shielding; based on the zero-field-cooled data in Fig. 1 we extract a residual magnetic field of −35 μT.

We calibrate the magnetic field at the sample position using two complementary methods (cf. Fig. S2). At low fields, $B < 100$ μT, we exploit the flux periodicity of a Gralmonium flux qubit, set by the qubit loop area of 94 μm^2. The qubit is identical in design to Ref. [5] and is mounted in the same sample holder, instead of the grAl resonators discussed in the main text. At fields $B > 100$ mT, calibration is performed using electron spin resonance (ESR) of $g = 2$ spin−1/2 paramagnetic impurities in grAl resonators, commonly observed in the environment of superconducting devices [3, 4]. When the spins are tuned into resonance with the grAl resonator ($f_\mathrm{r} = 7.627$ GHz), we observe enhanced microwave losses, manifesting as a dip in the internal quality factor Q_i at a magnetic field $B = hf_\mathrm{r}/g\mu_\mathrm{B} = 272.5$ mT (cf. Fig. S2 bottom inset). Following Ref. [3], to minimize vortex trapping and resonator frequency shift during this sweep, the chip is aligned parallel to the magnetic field.

II. REPEATED INITIALIZATION OF VQS

We measured VQs in six grAl resonators (samples A - F) fabricated on two wafers. Sample A is discussed in the main text. Samples C - F were deposited in a different e-beam evaporator than samples A and B, yielding a sheet resistance of $R_{\square} = 1.2\,\mathrm{k}\Omega/\square$ for a film-thickness of 30 nm. Although all resonators were nominally 3 µm wide, AFM measurements revealed post-fabrication variations: resonators A and B had slanted edges with an average width of 3.15 µm (cf. Extended Data Figure. 1), whereas resonators C - F featured sharp edges and a measured width of 2.8 µm.

Figure S3 summarizes field-cooling statistics for sample C, chosen to minimize width-related uncertainty that propagates to the field cooling bias ϕ/ϕ_{S}. We performed a total of 100 cooling cycles, one reference zero field and 99 at four bias fields, $\phi/\phi_{\mathrm{S}} \in \{0.75,\ 0.9,\ 1 \text{ and } 1.1\}$, where ϕ_{S} corresponds to $B_{\mathrm{cd}} = 0.93\,\mathrm{mT}$. Each cycle consists of a warmup to $T > 10\,\mathrm{K} > T_{\mathrm{c}}$ followed by cooling to $T < 50\,\mathrm{mK}$. Fieldsweeps taken near base temperature fall into three categories. At $\phi/\phi_{\mathrm{S}} = 0.75$, we consistently observe no VQs coupled to the readout resonator (cf. Fig. S3a): the resonator shows neither avoided level crossings nor a frequency shift relative to a zero-field-cooled reference (representative sweep in Fig. S3b). For $\phi/\phi_{\mathrm{S}} \geq 0.9$ the response changes qualitatively (Fig. S3c-f). We assign a VQ count of one to sweeps exhibiting avoided level crossings consistent with a single VQ coupled to the resonator (Fig. S3c), as in the main text Fig. 1d. In all other cases, we assign a VQ count $\neq 1$, because the number of coupled VQs cannot be determined unambiguously. These cases include discrete frequency jumps in the resonator response (Fig. S3d), a frequency shift relative to the zero-field-cooled reference (Fig. S3e), and multiple avoided level crossings (Fig. S3f), which may indicate more than one coupled VQ. Although we attribute all these signatures to vortices in the grAl resonator, an unambiguous identification would require two-tone spectroscopy as in Fig. 1e. Note that for all measurements with VQ count = 0 or 1 shown in Fig. S3, we observe no hysteresis between two consecutive sweeps taken in opposite directions.

Figure S4a shows VQ field spectra from four field-cooling (FC) preparation cycles, each involving thermal cycling of the grAl resonator to $T > 10\,\mathrm{K} > T_{\mathrm{c}}$ followed by field cooling at $B_{\mathrm{cd}} = 820\,\mu\mathrm{T}$. We reproducibly observe hyperbolic VQ spectra with varying sweet spot frequency and eccentricities. Similar results are obtained by field cooling other grAl resonator samples (cf. Fig. S4b). Interestingly, VQs can also be introduced by zero-field-cooling (ZFC) the grAl resonator, followed by applying a field $B \approx 4\,\mathrm{mT}$ at base temperature. As shown in Fig. S4c and Fig. S4d, the resulting spectra for VQs induced in ZFC samples exhibit hyperbolic characteristics comparable to those from FC. Previous observations of vortex trapping in ZFC grAl resonators (Ref. [3]) can now be suspected to also give rise to VQs.

Including the sample characterized in the main text, we measured a total of 32 VQ-resonator spectra prepared via field cooling across samples A-F. Multiple field-cooling cycles were performed with B_{cd} varied in the vicinity of the threshold field. In Fig. S5, we show the extracted VQ-resonator characteristics and find no correlation with the cool-down field.

III. RABI OSCILLATION AND ACTIVE STATE PREPARATION

Figure S6 shows Rabi oscillations of the excited-state population, confirming coherent quantum TLS operation. While thermal initialization yields a population inversion of 70%, conditional π-pulses enable ground- and excited-state preparation fidelities of 96.8 % and 91.7 %, limited by readout fidelity.

IV. DISPERSIVE MEASUREMENTS

In Fig. S7 we show IQ histograms used to extract the dispersive shift χ (cf. Fig 2 and Fig. S10). The two IQ clouds corresponding to $|g\rangle$ and $|e\rangle$ rotate along the S_{11} circle in the IQ plane, with a radius set by the square root of the measurement photon number [7, 8]. The absence of additional clouds indicates a well-isolated computational qubit basis.

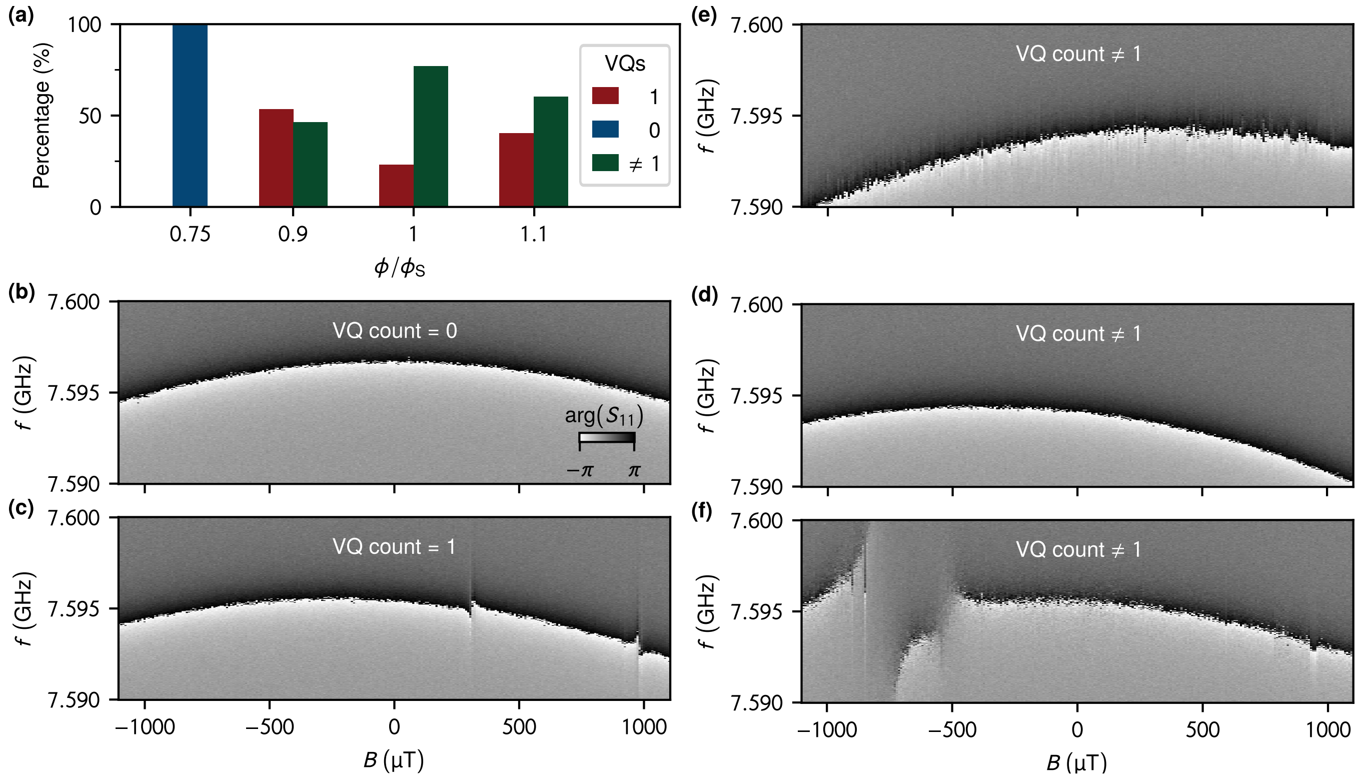

Figure S3. **Field-cooling statistics for a total of 99 cooldowns.** **(a)** Histogram of normalized VQ counts versus bias fields ϕ/ϕ_S. **(b - f)** Representative fieldsweeps illustrating the VQ-count classification are shown as follows: zero VQs in (b), one VQ in (c), and various other situations where vortices are trapped but the VQ count cannot be determined unambiguously in (d - f).

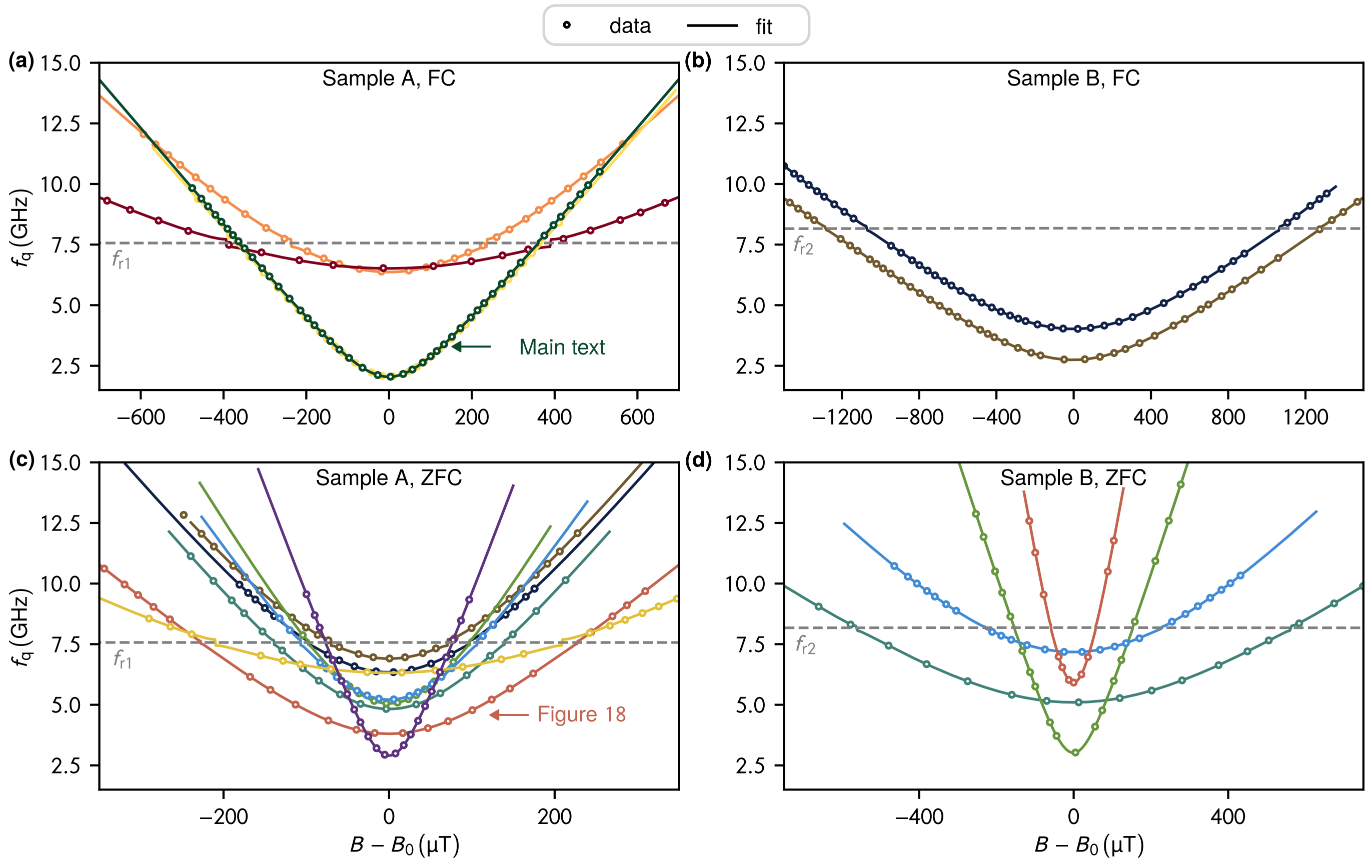

Figure S4. **Spectroscopy of additional VQs.** Extracted VQ frequency with fits to the Hamiltonian (Eq. 2) as a function of magnetic field detuning from their respective sweet spots B_0, which differ between preparation cycles (cf. Fig. S5b). Different colors indicate independent preparation cycles. Data are shown for two resonators fabricated from the same grAl film and identical geometry: Sample A (**a,c**, $f_{r1} = 7.5\,\mathrm{GHz}$) and Sample B (**b,d**, $f_{r2} = 8.1\,\mathrm{GHz}$). VQs are introduced by field cooling (top panels) or by zero-field cooling followed by ramping to $1\,\mathrm{mT}$ (bottom panels). Gray dashed lines mark the corresponding bare resonator frequencies.

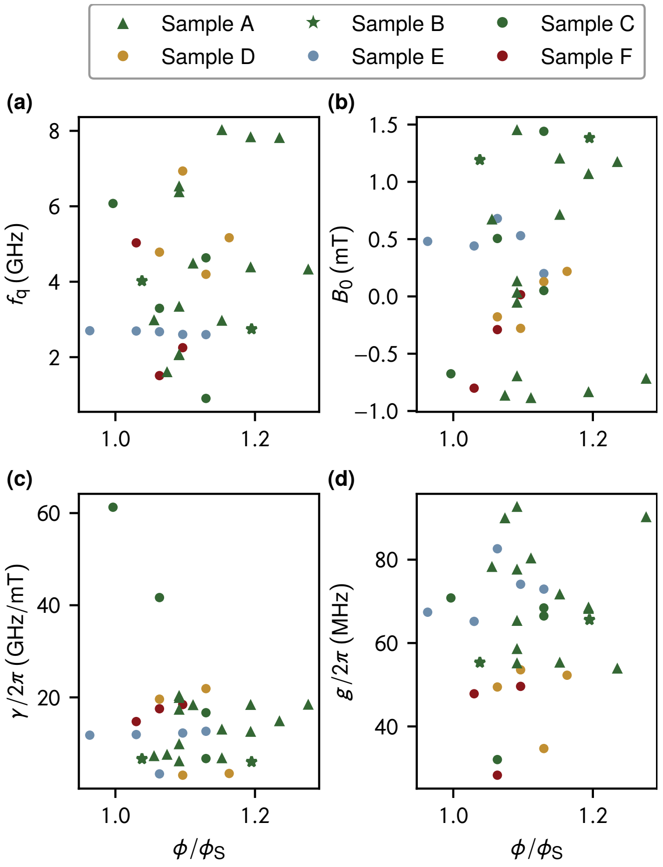

Figure S5. **Dependence of VQ parameters on bias field.** **(a)** Sweet-spot qubit frequency f_q, **(b)** offset field B_0, **(c)** gyromagnetic ratio γ, and **(d)** VQ-resonator coupling g as a function of the field bias ϕ during field cooling, normalized to ϕ_S (cf. main text and Ref. [6]). The values ϕ/ϕ_S are given by the cooldown field and each sample's respective width. Parameters are extracted from fits to Eq. 2. Spectroscopy for Samples A and B is shown in Fig. S4. Samples C – F were fabricated from a grAl film deposited in a different e-beam evaporator compared to the rest. Samples A – C have the same dimensions (cf. Extended Data Figure. 1), while the resonator length increases from 400 µm (Sample C) to 720 µm (Sample F).

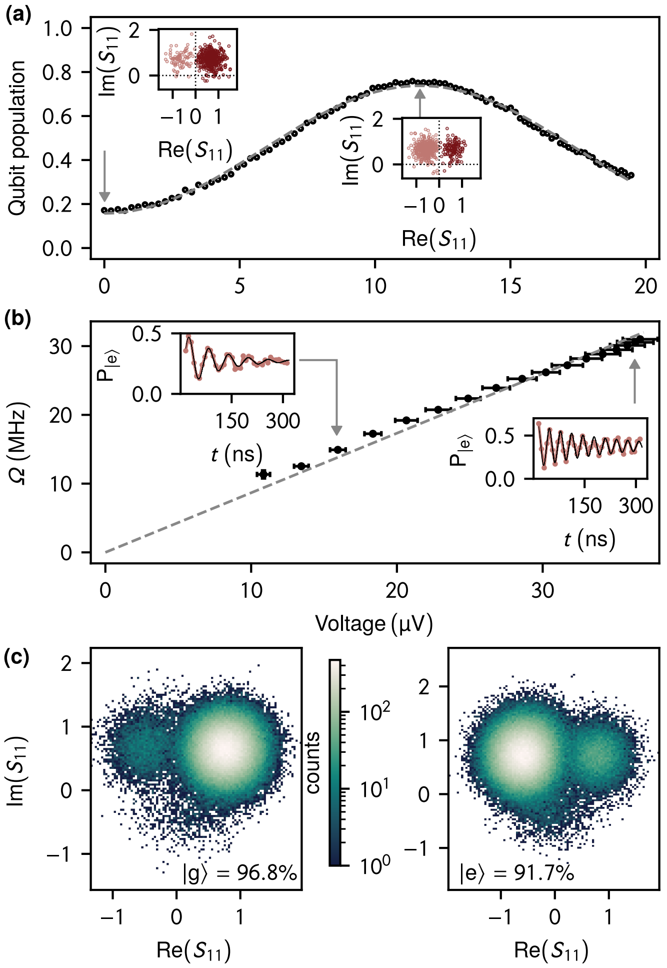

Figure S6. **Coherent control of the VQ.** **(a)** Rabi oscillations of the excited-state population (black circles) as a function of the drive amplitude at the sample holder. **Insets:** Scatter plots of repeated S_{11} measurements in the complex plane, normalized to the mean $|S_{11}(|g\rangle)|$, show population inversion of the ground (dark red) and excited (light red) state population between drive amplitudes of 0 µV and 25 µV (left and right inset, respectively). **(b)** Extracted Rabi frequency Ω (black circles) versus drive amplitude with a linear fit (gray line). Error bars indicate the uncertainty in the power delivered to the sample holder. **Insets:** Rabi oscillations of the excited-state population $P_{|e\rangle}$ as a function of pulse duration t for rectangular pulses with amplitudes of 16 µV (left inset) and 36.3 µV (right inset). Black lines indicate fits to a damped cosine function. **(c)** IQ histograms of 2×10^5 single-shot active state preparations in the ground (left) and excited (right) states, using conditional π-pulses triggered on detection of $|e\rangle$ or $|g\rangle$, respectively.

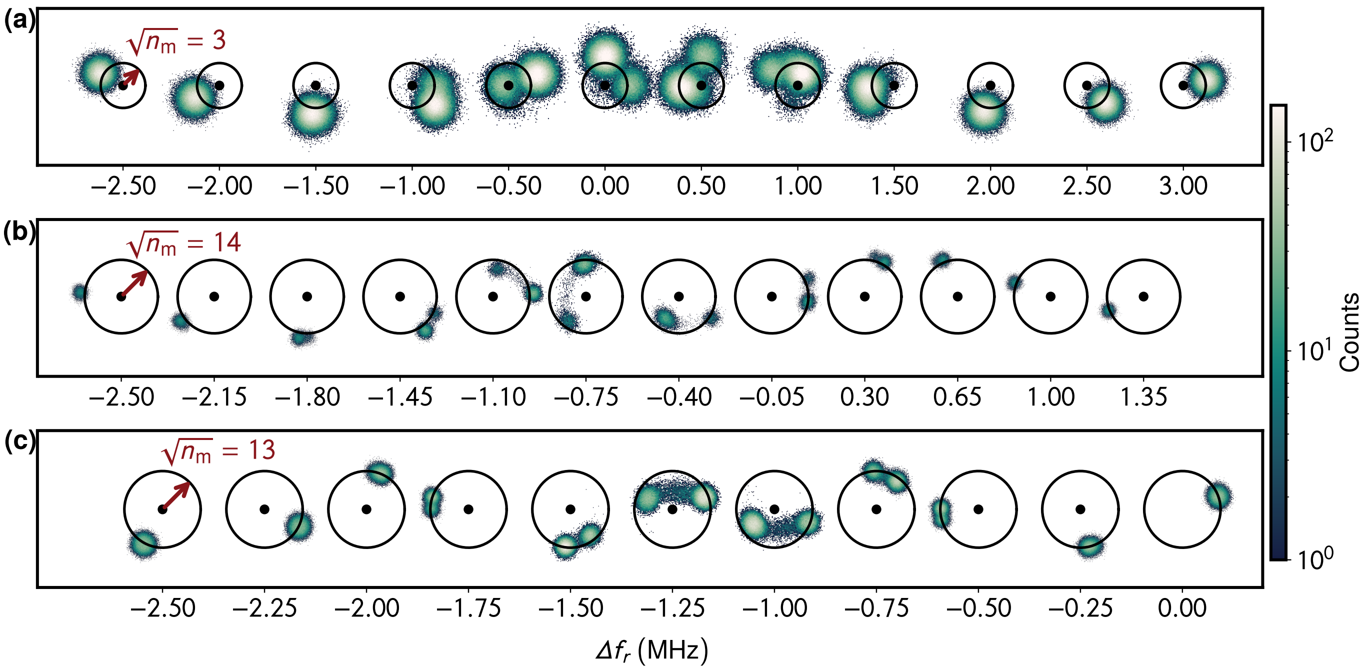

Figure S7. **IQ histograms versus readout detuning.** IQ histograms, plotted as a function of readout detuning Δf_r from the resonator frequency. Black circles indicate the complex resonator response for a readout pulse corresponding to n_m measurement photons. Measurements are shown for **(a)** the FC VQ in Sample A (main text VQ), **(b)** the VQ induced in ZFC Sample A (cf. Fig. S4), and **(c)** the FC VQ in Sample C (cf. Fig. S5).

V. ASYMMETRIC QUANTUM RABI MODEL

In the following, we justify the specific form of the AQRM in Eq. 2 used to model the qubit-resonator system. To reproduce the observed hyperbolic qubit spectrum, two orthogonal field components are required: a pseudo-field $\tilde{\boldsymbol{B}}$ and an applied field $\boldsymbol{B}'$ measured from the sweet spot, yielding a qubit Hamiltonian $\mathcal{H}_{\mathrm{q}} = \gamma\boldsymbol{S}\cdot(\tilde{\boldsymbol{B}}+\boldsymbol{B}')$. Without loss of generality, we assume coupling along the qubit's x-axis, leading to the interaction $\mathcal{H}_{\mathrm{c}} = \hbar g(\hat{a}^\dagger+\hat{a})\sigma_x$. The orthogonality $\tilde{\boldsymbol{B}} \perp \boldsymbol{B}'$ inherently introduces a transverse coupling component, manifesting experimentally as avoided level crossings (cf. Fig. 1d). The full AQRM thus reads:

$$\mathcal{H}_{\mathrm{AQRM}} = \mathcal{H}_{\mathrm{r}} + \mathcal{H}_{\mathrm{c}} + \gamma\boldsymbol{S}\cdot\tilde{\boldsymbol{B}} + \gamma\boldsymbol{S}\cdot\boldsymbol{B}'. \tag{1}$$

We parametrize the fields via angles θ and ϕ:

$$\tilde{\boldsymbol{B}} = \tilde{B}\begin{pmatrix}\cos\theta\\ 0\\ \sin\theta\end{pmatrix}, \tag{2}$$

$$\boldsymbol{B}' = B'\begin{pmatrix}-\sin\phi\,\sin\theta\\ \cos\phi\\ \sin\phi\,\cos\theta\end{pmatrix}, \tag{3}$$

as indicated in Fig. S8a. To match the observed avoided crossings at symmetric values of $\boldsymbol{B}'$ around the sweet spot, the magnitude of the projection of the effective field $\boldsymbol{B}_{\mathrm{eff}} = \boldsymbol{B}' + \tilde{\boldsymbol{B}}$ onto the coupling axis x must remain invariant under $\boldsymbol{B}' \to -\boldsymbol{B}'$. This limits the model to $\phi = 0$ for all θ, except at $\theta = 0$ and $\theta = \pi/2$, where $\phi \in [0, \pi)$.

Among the resulting scenarios, we focus on three representative cases (cf. Fig. S8b-d), jointly fitting both the VQ and resonator frequency dependence (cf. Fig. 1d,e) in field. All other configurations can be mapped onto these three.

In the configuration where the pseudo-field aligns with the coupling axis (cf. Fig. S8b), $\boldsymbol{B}'$ lies in the yz-plane. For $\phi = 90°$ the Hamiltonian becomes:

$$\mathcal{H}_{\{0°,90°\}} = \mathcal{H}_{\mathrm{r}} + \mathcal{H}_{\mathrm{c}} + \frac{\hbar\gamma}{2}\sigma_x\cdot\tilde{B} + \frac{\hbar\gamma}{2}\sigma_z\cdot B'$$

This corresponds to pure longitudinal coupling at the sweet spot, resulting in zero dispersive shift [9], in contrast to the experimentally observed χ.

Figure S8c illustrates the case of pure transverse coupling, with both fields orthogonal to the coupling axis. The Hamiltonian

$$\mathcal{H}_{\{90°,0°\}} = \mathcal{H}_{\mathrm{r}} + \mathcal{H}_{\mathrm{c}} + \frac{\hbar\gamma}{2}\sigma_z\cdot\tilde{B} + \frac{\hbar\gamma}{2}\sigma_y\cdot B'$$

can be mapped to the SQRM (cf. Eq. 1) and fails to capture the non-monotonic dependence of χ (cf. yellow line in Fig. S8e).

The best agreement with the measured dispersive shift is achieved in the configuration shown in Fig. S8d, where the applied magnetic field is aligned with the coupling axis. This introduces a longitudinal component in the interaction away from the sweet spot and gives the Hamiltonian:

$$\mathcal{H}_{\{90°,90°\}} = \mathcal{H}_{\mathrm{r}} + \mathcal{H}_{\mathrm{c}} + \frac{\hbar\gamma}{2}\sigma_z\cdot\tilde{B} - \frac{\hbar\gamma}{2}\sigma_x\cdot B',$$

identical to Eq. 1 used to fit the data in the main text.

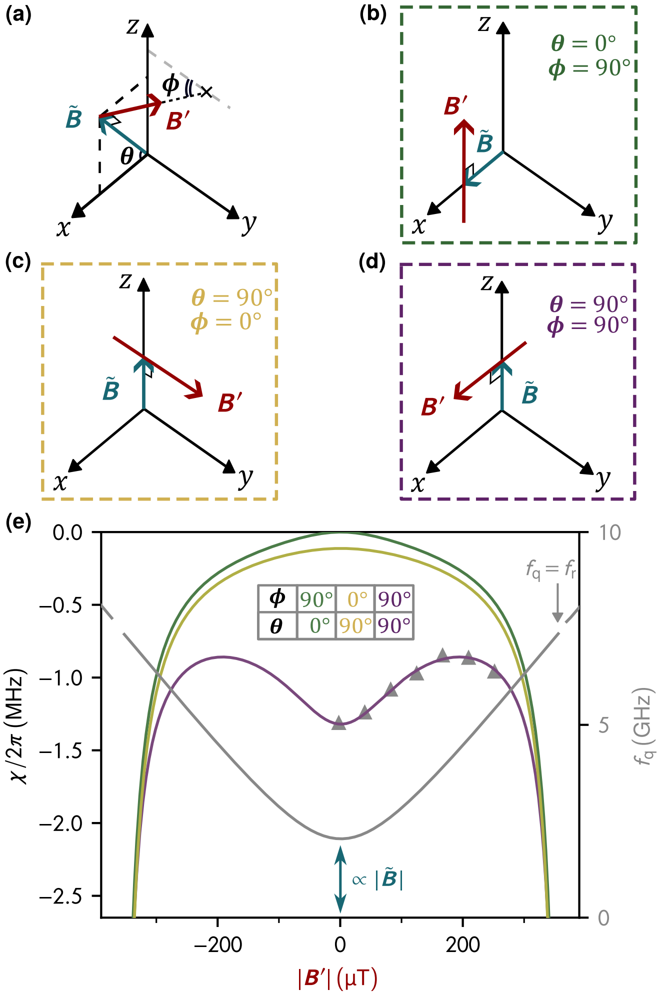

Figure S8. **Field orientations in the AQRM Hamiltonian.** **(a)** Coordinate system defining the orientation of the pseudo-field $\tilde{\boldsymbol{B}}$ and the applied field relative to the sweet spot $\boldsymbol{B}'$ for the σ_x interaction in the AQRM (Eq. (1)). The pseudo-field $\tilde{\boldsymbol{B}}$ lies in the xz-plane at angle θ to the x-axis, and is orthogonal to $\boldsymbol{B}'$, which is tilted at an angle ϕ to the y-axis. Note that z and x coordinates here do not coincide with the spatial directions of the field in the laboratory frame. **(b-d)** Representative orientations of $\tilde{\boldsymbol{B}}$ and $\boldsymbol{B}'$ used to model the VQ-resonator system via the AQRM (Eq. (1)). All other configurations can be mapped onto or constructed from these cases. **(d)** Measured dispersive shift χ (triangle markers, left axis) and qubit frequency f_{q} (gray line, right axis) as a function of $|\boldsymbol{B}'|$. The predicted χ for the field parameterizations (ϕ, θ) of the AQRM in panels (b-d) are indicated as lines in green, yellow, and purple. Data for χ and f_{q} are identical to Fig. 2d.

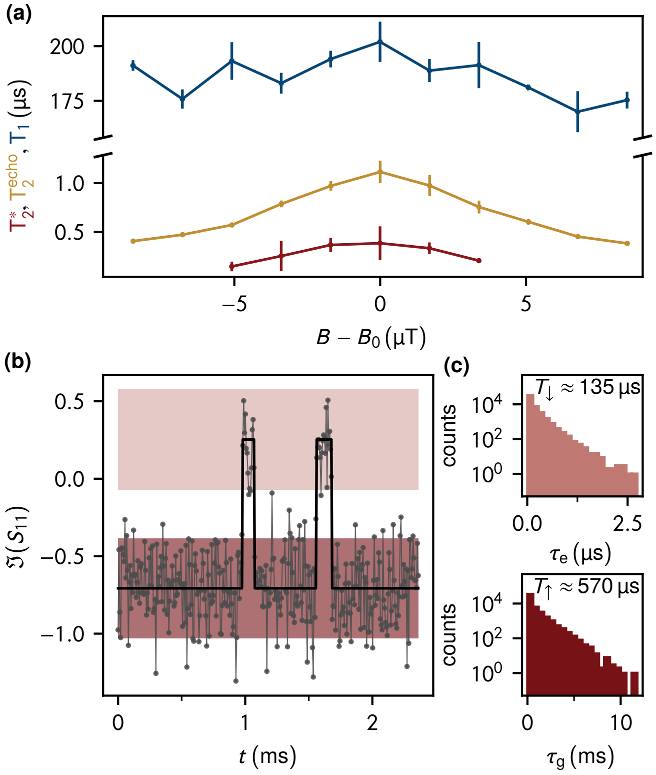

Figure S9. **Extended time domain characterization for the main text VQ.** **(a)** Energy relaxation time T_1 (blue), Ramsey coherence time T_2^* (red), and Hahn echo coherence times T_2^{echo} (yellow) as a function of the applied magnetic field detuning from the sweet spot $B - B_0$. Markers and error bars denote the mean and standard error of the mean of two consecutive field sweeps, each averaging 5000 single-shot measurements. **(b,c)** Quantum jump detection of the VQ at the sweet spot B_0. **(b)** Representative time trace of the imaginary part of the resonator reflection coefficient $\Im(S_{11})$ (gray markers). Qubit states are assigned using a latching filter (black line) based on 1.5 standard deviation thresholds (dark red band for $|g\rangle$, light red for $|e\rangle$) centered on their respective means. **(c)** Histograms of dwell times in the ground (lower panel) and excited (upper panel) states from 5×10^6 single-shot measurements. Average excitation and relaxation times are $T_\uparrow = 570\,\mu\text{s}$ and $T_\downarrow = 135\,\mu\text{s}$ respectively.

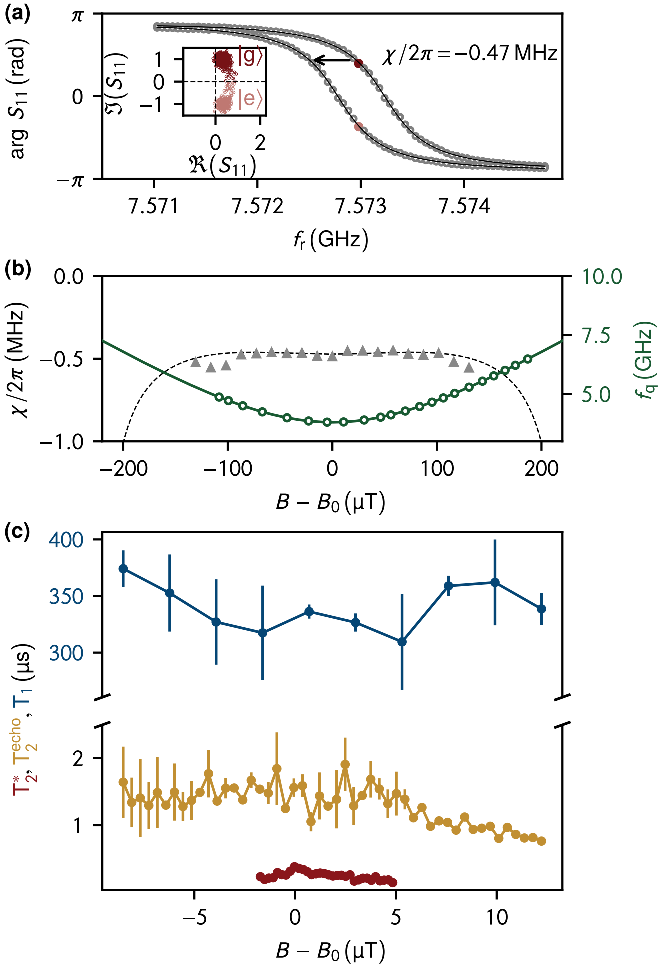

Figure S10. **Characterization of a VQ introduced in a ZFC sample.** The VQ is introduced by ZFC of the grAl resonator followed by ramping to 1 mT (cf. Fig. S4c). **(a)** Dispersive shift measurement (similar to Fig. 2b), yielding $\chi = -0.47\,\text{MHz}$. Dark red ($|g\rangle$) and light red ($|e\rangle$) markers denote data extracted from the IQ clouds shown in the inset. **Inset:** Normalized scatter plot of repeated S_{11} measurements in the complex plane, referenced to the ground state amplitude. Note that additional IQ cloud histograms for this VQ are presented in Fig. S7. **(b)** Measured dispersive shift χ (triangles) near B_0 (similar to Fig. 2d). The green line is a fit of the qubit spectrum (right axis) to Eq. 1 while the dashed line indicates the predicted χ-dependence. **(c)** Extracted energy relaxation time T_1 (blue), and coherence times T_2^* (red) and T_2^{echo} (yellow) as a function of detuning from B_0 (similar to Fig. S9a). Markers and error bars represent the mean and standard deviation of two consecutive field sweeps.

VI. EXTENDED TIME DOMAIN CHARACTERIZATION AND QUANTUM JUMPS OF VQS

In Fig. S9a, we present energy relaxation and coherence of the VQ as a function of magnetic field detuning from the sweet spot. Whereas T_1 remains largely insensitive to detuning, coherence times decrease away from B_0, consistent with flux-noise-induced dephasing observed in superconducting flux qubits [5, 10–12]. In Fig. S9b, we use time-resolved S_{11} measurements to detect quantum jumps. We apply 1.2 µs long readout pulses, spaced 5 µs apart and corresponding to an average photon number $\overline{n}_r = 7$, similar to the main-text measurements (cf. Fig. 3). State transitions are identified with a two-point latching filter, which registers a

jump when $\Im(S_{11})$ enters the 1.5 × standard deviation band centered on the mean of the $|g\rangle$ or $|e\rangle$-states, as indicated by the shaded bands. Dwell-time histograms (Fig. S9c) give average excitation and relaxation times of $T_\uparrow = 570\,\mu s$ and $T_\downarrow = 135\,\mu s$, corresponding to a T_1 time of $T_1 = (T_\downarrow^{-1} + T_\uparrow^{-1})^{-1} = 110\,\mu s$, within the temporal fluctuations of the free decay time shown in the main text Fig. 3a.

Figure S10 provides a complete characterization of a VQ introduced after ZFC, yielding results consistent with those of the main-text sample. We measure a dispersive shift for this VQS-resonator system of $\chi/2\pi = 0.5\,\mathrm{MHz} < \kappa/2\pi = 0.75\,\mathrm{MHz}$ with a magnetic field dependence away from the sweet spot that aligns with the AQRM model (Fig. S10b). Moreover, we observe energy relaxation and coherence times (Fig. S10c) comparable to those in FC VQs.

VII. MAGNETIC FIELD DISPERSION FROM A VORTEX TUNNELING MODEL

To model VQ states we focus on the minimal configuration that yields strong anharmonicity: a double-well potential formed by two closely spaced pinning sites, allowing significant wavefunction delocalization. The applied magnetic field modulates this potential through the Gibbs free energy of the vortex, which depends linearly on B (cf. Eq. 3). A quantum mechanical model of vortex tunneling is developed to characterize the qubit's frequency, magnetic field dispersion, and anharmonicity.

The Gibbs free energy from Eq. 3 (cf. [13, 14]) is $\hat{G}_1 = \hat{V}_\mathrm{M} + \hat{V}_\mathrm{SE}$, where the Meissner term $\hat{V}_\mathrm{M}$ arises from the interaction between a Pearl vortex, at position x along the wire width, and screening currents induced by a perpendicular magnetic field B. The term $\hat{V}_\mathrm{SE}$, independent of magnetic field, gives the self energy due to the vortex current. Assuming the vortex is far from the film edges along the wire length (y-direction),

$$\hat{V}_\mathrm{M} = 2\pi\varepsilon_0 \left(\frac{B - n\Phi_0}{\Phi_0}\right) \hat{x}\,(\hat{x} - w)\,, \qquad (4)$$

where n is the surface density of vortices, and

$$\hat{V}_\mathrm{SE} = \varepsilon_0 \ln\left[\frac{2w}{\pi\xi} \sin\left(\frac{\pi\hat{x}}{w}\right) + 1\right]. \qquad (5)$$

We model the two pinning sites using two-dimensional Lorentzian potentials:

$$\hat{V}_\mathrm{pin} = -\sum_{i=1,2} V_i \left(1 + \frac{(\hat{x} - x_i)^2 + (\hat{y} - y_i)^2}{\sigma_i^2}\right)^{-1} \qquad (6)$$

where V_i, σ_i, and (x_i, y_i) denote the depth, width, and position of each pinning site, respectively. Therefore the total potential energy is:

$$\hat{V} = \hat{V}_\mathrm{M} + \hat{V}_\mathrm{SE} + \hat{V}_\mathrm{pin}. \qquad (7)$$

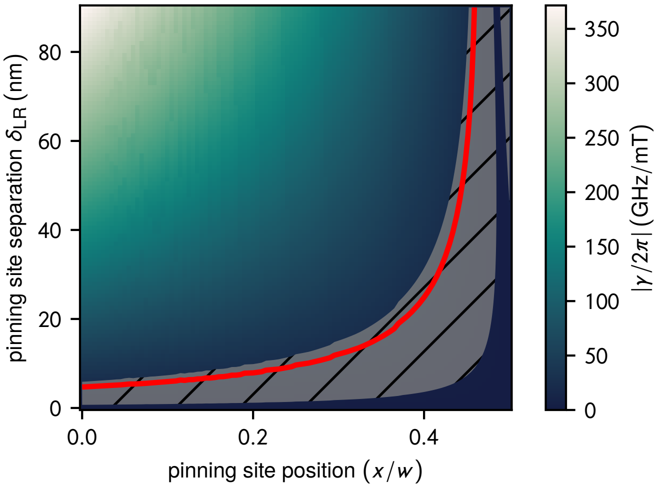

Figure S11. **VQ magnetic field dispersion.** Calculated gyromagnetic ratio γ (Eq. 2) as a function of the separation δ_LR between two pinning sites forming the double-well potential, plotted against the position of the first site along the resonator width. Following Eq. (10), γ quantifies the tunability of the double-well spectrum with external magnetic field B, i.e., the slope of the hyperbolic qubit spectrum. The hatched area indicates the range of $\gamma = 3$–$25\,\mathrm{GHz/mT}$ values extracted from Hamiltonian fits across all measured VQ–resonator systems (cf. Fig. S5c). The red line indicates the measured $\gamma = 20\,\mathrm{GHz/mT}$ corresponding to the VQ in main text. It is important to note that although the Gibbs free energy favors a central position of the vortex ($x/w \approx 0.5$), in the presence of pinning its position can be significantly off-centered [15].

Following Eq. (7), the Hamiltonian for a pinned vortex of mass m_v is

$$\mathcal{H} = -\frac{\hbar^2}{2m_\mathrm{v}}\nabla^2 + \hat{V}(\hat{x}, \hat{y}; B). \qquad (8)$$

Due to the larger number of degrees of freedom of the microscopic model compared to the extracted parameters from the hyperbolic VQ spectrum, an unambiguous fit of the data to Hamiltonian Eq. (8) is not possible. Nevertheless, from the measured γ, B_0 and $\omega_q(B_0)$, we can constrain the separation between pinning sites δ_LR, the pinning potential asymmetry $V_1 - V_2$, and we can provide an estimate for the zero point fluctuation amplitude of the vortex in its pinning sites y_zpf, respectively. In the following, we discuss these quantities individually.

For a separation δ_LR between sites, the Gibbs energy difference is

$$\begin{aligned}\Delta G(x, \delta_\mathrm{LR}; B) &= |G_1(x - \delta_\mathrm{LR}/2, B) - G_1(x + \delta_\mathrm{LR}/2, B)| \\ &= |C(x, \delta_\mathrm{LR}) - h\gamma(x, \delta_\mathrm{LR})B| \qquad (9)\end{aligned}$$

where $x = (x_1 + x_2)/2$ with $x_{1,2}$ the positions of the two wells along the width of the resonator, and C is a constant independent of B. We can identify

$$h\gamma = 2\pi\left(\frac{\varepsilon_0}{\Phi_0}\right)|\delta_\mathrm{LR}(2x - w)|, \qquad (10)$$

as the gyromagnetic ratio in the AQRM (cf. Eq. 2 and App. V), resulting in a hyperbolic VQ spectrum, corroborating the single-vortex model for the VQ.

In Fig. S11, the experimentally extracted range of $\gamma/2\pi = 3$–$25\,\mathrm{GHz/mT}$ (hatched region) indicates a large range of VQ locations where the pinning site separation is on the order of tens of nanometers, setting a microscopic length scale for the effective double-well potential, consistent with flux tunneling observed in granular aluminum nanojunction qubits (cf. Refs. [4, 5]). For each VQ, the measured γ imposes a constraint on $x = (x_1+x_2)/2$ in relation to $\delta_{LR} = |x_1-x_2|$, as shown by the line in Fig. S11, corresponding to the VQ discussed in the main text.

The measured B_0 imposes a second constraint, corresponding to the energy alignment of pinning sites $V(x_1, y_1; B_0)$ and $V(x_2, y_2; B_0)$. This takes the form

$$\frac{\hbar}{2}(\Omega_1 - \Omega_2) \approx (V_1 - V_2) + \Delta G(x, \delta_{\mathrm{LR}}; B_0), \tag{11}$$

with $\Omega_i = \sqrt{k_i/m_{\mathrm{v}}}$ the frequency corresponding to the curvature of the pinning potentials $k_i = 2V_i/\sigma_i^2$. For simplicity, we assume $k_1 = k_2 \equiv k$ and therefore, $\Omega_1 = \Omega_2 \equiv \Omega$, which gives

$$V_2 \approx V_1 + \Delta G(x, \delta_{\mathrm{LR}}; B_0). \tag{12}$$

Equation (12) sets the scale for the variability of pinning potential depths that give rise to VQs (cf. App. II).

Finally, fitting $\omega_q(B_0)$ imposes a constraint on the lowest two eigenenergies of Hamiltonian $\mathcal{H}(B_0)$,

$$\mathcal{H}(B_0) = \hbar\Omega\left[-y_{\mathrm{zpf}}^2\nabla^2 + \frac{\hat{V}(\hat{x}, \hat{y}; B_0)}{\hbar\Omega}\right], \tag{13}$$

where we have introduced the characteristic zero point fluctuation $y_{\mathrm{zpf}} = \sqrt{\hbar/2m_{\mathrm{v}}\Omega} = ((8V_i m_{\mathrm{v}})^{0.25}/\sqrt{\hbar\sigma_i})^{-1}$. In principle, one could numerically solve the time-independent Schrödinger equation corresponding to Eq. (13) to fit y_{zpf}. However, this approach would require specific assumptions about the shape of the pinning potential, i.e., the microscopic pinning mechanism, which remains uncertain. While a promising direction for future research, this is beyond the scope of the present work. For now, we simply argue that to observe significant qubit frequencies in the GHz range at B_0, y_{zpf} must be in the range $\sigma \lesssim y_{\mathrm{zpf}} \lesssim \delta_{\mathrm{LR}}$. As we will show in the next section, this estimate proves useful for evaluating the VQ-resonator coupling strength.

VIII. VQ-RESONATOR INTERACTION

An unambiguous description of the VQ-resonator coupling mechanism would require a detailed microscopic understanding of the VQ, which is currently lacking. Instead, we present here an order-of-magnitude estimate. When a vortex tunnels between two pinning sites inside the resonator, the spatial distribution of its circulating current is redistributed. Following the approach of Ref. [16], the resulting change in vortex energy due to its overlap with the resonator current gives the interaction energy

$$U_{\mathrm{int}} = \Phi_0 I_{\mathrm{zpf}} \frac{\delta_{\mathrm{LR}}}{w} \tag{14}$$

where $\delta_{\mathrm{LR}} \sim 10\,\mathrm{nm}$ characterizes the effective x-displacement of the vortex supercurrent distribution between the two pinning sites (as estimated in App. VII). Note that this equation holds for both Abrikosov and Pearl vortices. We can express the current as $I_{\mathrm{zpf}} = \sqrt{\frac{\hbar\omega_{\mathrm{r}}}{2L_{\mathrm{r}}}}$, where $L_{\mathrm{r}} = 63\,\mathrm{nH}$ and $\omega_{\mathrm{r}} = 2\pi \times 7.5\,\mathrm{GHz}$ are the resonator inductance and frequency, respectively. The resulting coupling energy scale is

$$\frac{\tilde{g}}{2\pi} = \frac{U_{\mathrm{int}}}{h} \approx 130\,\mathrm{MHz}, \tag{15}$$

corresponding to $\tilde{g}/\omega_{\mathrm{r}} \simeq 1\,\%$, comparable with the experimentally extracted coupling strengths reported in Fig. S5d.

IX. VORTEX-VORTEX INTERACTION

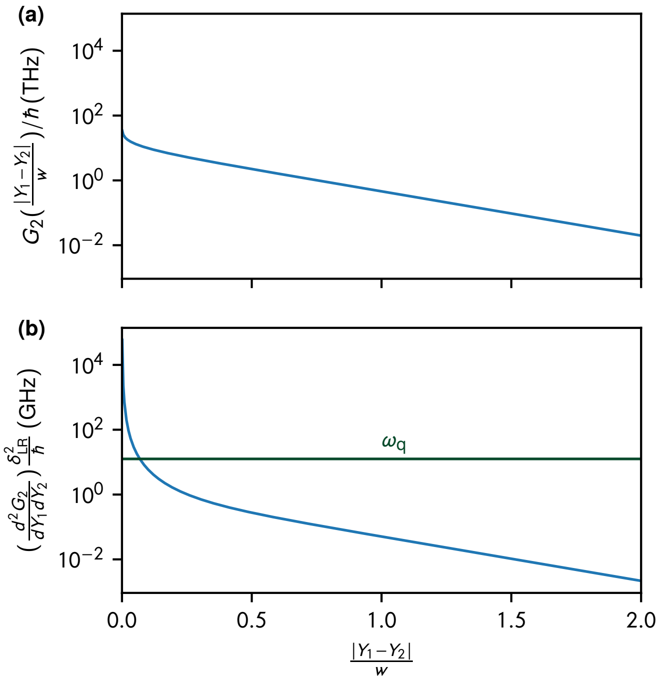

Figure S12. **Interaction between vortices.** **(a)** Mutual interaction potential G_2 between two vortices located along the center of the wire ($X_1 = X_2 = w/2$) as a function of their separation $|Y_1 - Y_2|$. **(b)** Estimated qubit–qubit interaction strength between two vortex-qubits pinned at positions $X_1 = X_2 = w/2$, versus their separation along the length of the resonator $|Y_1 - Y_2|$, for $\delta_{\mathrm{LR}} = 10\,\mathrm{nm}$. The green line indicates one of the measured qubit frequencies ω_{q}.

Field cooling above $\phi \gtrsim \phi_{\mathrm{s}}$ induces a large number of vortices, which may become trapped at various pinning

sites along the wire. To assess the impact of vortex-vortex interaction on the dynamics of the vortex-qubit, we derive the interaction Hamiltonian $\mathcal{H}_{\rm int}$ between two pinned vortices. We begin with the Gibbs free energy due to interaction between two Pearl vortices ($i = \{1, 2\}$), located at positions $\boldsymbol{r}_i = (x_i, y_i)$ [13]:

$$G_2(\boldsymbol{r}_1, \boldsymbol{r}_2) = \varepsilon_0 \ln\left(\frac{\cosh(\frac{\pi(y_1-y_2)}{w}) - \cos(\frac{\pi(x_1+x_2)}{w})}{\cosh(\frac{\pi(y_1-y_2)}{w}) - \cos(\frac{\pi(x_1-x_2)}{w})}\right) \tag{16}$$

where $\varepsilon_0 = \Phi_0^2/(2\pi\mu_0\Lambda)$. Here, the wire geometry is defined over $y \in [0, L]$ along its length and $x \in [0, w]$ along its width. This interaction is repulsive and decays exponentially over length scales on the order of w, as shown in Fig. S12a.

In the local qubit basis, the position operator can be expressed as:

$$\hat{r}_i^{(\xi)} = \delta_{\rm LR}\left(\alpha_i^{(\xi)}\hat{\sigma}_x + \beta_i^{(\xi)}\hat{\sigma}_z\right)$$

where $\xi \in \{x, y\}$, $\delta_{\rm LR}$ is the length scale of vortex tunneling, and the coefficients $\alpha_i^{(\xi)}$ and $\beta_i^{(\xi)}$ are dependent on the pinning site geometry, dimensionless, and typically of the order unity.

Assuming the vortex separation is large compared to the tunneling distance, $|\boldsymbol{R}_1 - \boldsymbol{R}_2| \gg \delta_{\rm LR}$, we linearize the interaction to obtain the effective Hamiltonian:

$$\mathcal{H}_{\rm int}(\boldsymbol{r}_1, \boldsymbol{r}_2) = \hat{r}_1^T [(\boldsymbol{\nabla}_{\boldsymbol{R}_1}\boldsymbol{\nabla}_{\boldsymbol{R}_2})\, G_2(\boldsymbol{R}_1, \boldsymbol{R}_2)]\ \hat{r}_2, \tag{17}$$

where $(\boldsymbol{\nabla}_{\boldsymbol{R}_1}\boldsymbol{\nabla}_{\boldsymbol{R}_2})\, G_2(\boldsymbol{R}_1, \boldsymbol{R}_2)$ is the 2×2 mixed-derivatives Hessian matrix of $G_2(\boldsymbol{R}_1, \boldsymbol{R}_2)$, defined as:

$$[(\boldsymbol{\nabla}_{\boldsymbol{R}_1}\boldsymbol{\nabla}_{\boldsymbol{R}_2})\, G_2(\boldsymbol{R}_1, \boldsymbol{R}_2)]_{\xi,\xi'} = \frac{\partial^2 G_2}{\partial R_1^{(\xi)} \partial R_2^{(\xi')}},$$

for $\{\xi, \xi'\} \in \{x, y\}$. To estimate the typical qubit-qubit coupling strength, we consider a simplified geometry where both vortex centers lie along the central axis of the wire and are aligned along the y-direction. Under this condition, the only non-zero component of the interaction tensor is $[(\boldsymbol{\nabla}_{\boldsymbol{R}_1}\boldsymbol{\nabla}_{\boldsymbol{R}_2})\, G_2(\boldsymbol{R}_1, \boldsymbol{R}_2)]_{y,y}$. The resulting interaction strength is plotted in Fig. S12b and compared to a representative qubit frequency $\omega_q = 12.5\,\text{GHz}$.

Due to the low vortex density generated during field cooling ($N_\odot \simeq 1$), it is unlikely that two vortices become trapped at separations significantly smaller than w. In this case, the vortex-vortex interaction strength is at least one order of magnitude smaller than the typical vortex-qubit frequency (cf. Fig. S12). Moreover, since $\omega_{\rm q}$ depends exponentially on the tunneling barrier, small variations in local pinning geometries can lead to detunings comparable to or larger than the qubit frequency. This further suppresses coherent coupling between neighboring vortex qubits. These facts support the interpretation that a single pinned vortex accounts for the experimental observations presented in the main text.

[1] R. Rehammar and S. Gasparinetti, Low-pass filter with ultra-wide stopband for quantum computing applications, IEEE Transactions on Microwave Theory and Techniques **71**, 3075–3080 (2023).

[2] P. Winkel, I. Takmakov, D. Rieger, L. Planat, W. Hasch-Guichard, L. Grünhaupt, N. Maleeva, F. Foroughi, F. Henriques, K. Borisov, J. Ferrero, A. V. Ustinov, W. Wernsdorfer, N. Roch, and I. M. Pop, Nondegenerate parametric amplifiers based on dispersion-engineered josephson-junction arrays, Phys. Rev. Appl. **13**, 024015 (2020).

[3] K. Borisov, D. Rieger, P. Winkel, F. Henriques, F. Valenti, A. Ionita, M. Wessbecher, M. Spiecker, D. Gusenkova, I. M. Pop, and W. Wernsdorfer, Superconducting granular aluminum resonators resilient to magnetic fields up to 1 tesla, Applied Physics Letters **117**, 10.1063/5.0018012 (2020).

[4] S. Günzler, J. Beck, D. Rieger, N. Gosling, N. Zapata, M. Field, S. Geisert, A. Bacher, J. K. Hohmann, M. Spiecker, W. Wernsdorfer, and I. M. Pop, Spin environment of a superconducting qubit in high magnetic fields, Nat. Commun. **16**, 1 (2025).

[5] D. Rieger, S. Günzler, M. Spiecker, P. Paluch, P. Winkel, L. Hahn, J. K. Hohmann, A. Bacher, W. Wernsdorfer, and I. M. Pop, Granular aluminium nanojunction fluxonium qubit, Nature Materials **22**, 194–199 (2023).

[6] V. G. Kogan, Pearl's vortex near the film edge, Physical Review B **49**, 15874– (1994).

[7] M. Hatridge, S. Shankar, M. Mirrahimi, F. Schackert, K. Geerlings, T. Brecht, K. M. Sliwa, B. Abdo, L. Frunzio, S. M. Girvin, R. J. Schoelkopf, and M. H. Devoret, Quantum Back-Action of an Individual Variable-Strength Measurement, Science **339**, 178 (2013).

[8] U. Vool, I. Pop, K. Sliwa, B. Abdo, C. Wang, T. Brecht, Y. Gao, S. Shankar, M. Hatridge, G. Catelani, M. Mirrahimi, L. Frunzio, R. Schoelkopf, L. Glazman, and M. Devoret, Non-poissonian quantum jumps of a fluxonium qubit due to quasiparticle excitations, Physical Review Letters **113**, 247001 (2014).

[9] S. Richer and D. DiVincenzo, Circuit design implementing longitudinal coupling: A scalable scheme for superconducting qubits, Phys. Rev. B **93**, 134501 (2016).

[10] F. Yoshihara, K. Harrabi, A. O. Niskanen, Y. Nakamura, and J. S. Tsai, Decoherence of Flux Qubits due to $1/f$ Flux Noise, Phys. Rev. Lett. **97**, 167001 (2006).

[11] M. Stern, G. Catelani, Y. Kubo, C. Grezes, A. Bienfait, D. Vion, D. Esteve, and P. Bertet, Flux qubits with long coherence times for hybrid quantum circuits, Physical Review Letters **113**, 123601 (2014).

[12] F. Yan, S. Gustavsson, A. Kamal, J. Birenbaum, A. P. Sears, D. Hover, T. J. Gudmundsen, D. Rosenberg, G. Samach, S. Weber, J. L. Yoder, T. P. Orlando, J. Clarke, A. J. Kerman, and W. D. Oliver, The flux qubit revisited to enhance coherence and reproducibility, Nature Communications **7**, 12964 (2016).

[13] E. Bronson, M. P. Gelfand, and S. B. Field, Equilibrium configurations of pearl vortices in narrow strips, Physical Review B **73**, 144501 (2006).

[14] K. H. Kuit, J. R. Kirtley, W. Van Der Veur, C. G. Molenaar, F. J. G. Roesthuis, A. G. P. Troeman, J. R. Clem, H. Hilgenkamp, H. Rogalla, and J. Flokstra, Vortex trapping and expulsion in thin-film $YBa_2Cu_3O_{7-\delta}$ strips, Physical Review B **77**, 134504 (2008).

[15] R. Bai, A. Sepehri, Y.-L. Loh, A.-M. Valente-Feliciano, A. Herr, Q. Herr, and K. C. Nowack, Flux trapping in NbTiN strips and structures, arXiv preprint (2025), arXiv:2503.14457.

[16] L. N. Bulaevskii, M. J. Graf, C. D. Batista, and V. G. Kogan, Vortex-induced dissipation in narrow current-biased thin-film superconducting strips, Phys. Rev. B **83**, 144526 (2011).
